# Coronary computed tomography angiography (CCTA): effect of bolus-tracking ROI positioning on image quality

**DOI:** 10.1007/s00330-020-07131-x

**Published:** 2020-08-18

**Authors:** Heiner Nebelung, Thomas Brauer, Danilo Seppelt, Ralf-Thorsten Hoffmann, Ivan Platzek

**Affiliations:** Department of Radiology, Dresden University Hospital, Fetscherstr. 74, 01307 Dresden, Germany

**Keywords:** Computed tomography angiography, Coronary artery disease, Pulmonary artery, Signal-to-noise ratio, Propensity score

## Abstract

**Objectives:**

The aim of the study was to evaluate the effect of bolus-tracking ROI positioning on coronary computed tomography angiography (CCTA) image quality.

**Methods:**

In this retrospective monocentric study, all patients had undergone CCTA by step-and-shoot mode to rule out coronary artery disease within a cohort at intermediate risk. Two groups were formed, depending on ROI positioning (left atrium (LA) or ascending aorta (AA)). Each group contained 96 patients. To select pairs of patients, propensity score matching was used. Image quality with regard to coronary arteries as well as pulmonary arteries was evaluated using quantitative and qualitative scores.

**Results:**

In terms of the coronary arteries, there was no significant difference between both groups using quantitative (SNR AA 14.92 vs. 15.46; *p* = 0.619 | SNR LM 19.80 vs. 20.30; *p* = 0.661 | SNR RCA 24.34 vs. 24.30; *p* = 0.767) or qualitative scores (4.25 vs. 4.29; *p* = 0.672), respectively. With regard to pulmonary arteries, we found significantly higher quantitative (SNR RPA 8.70 vs. 5.89; *p* < 0.001 | SNR LPA 9.06 vs. 6.25; *p* < 0.001) and qualitative scores (3.97 vs. 2.24; *p* < 0.001) for ROI positioning in the LA than for ROI positioning in the AA.

**Conclusions:**

ROI positioning in the LA or the AA results in comparable image quality of CT coronary arteriography, while positioning in the LA leads to significantly higher image quality of the pulmonary arteries. These results support ROI positioning in the LA, which also facilitates triple-rule-out CT scanning.

**Key Points:**

*• ROI positioning in the left atrium or the ascending aorta leads to comparable image quality of the coronary arteries.*

*• ROI positioning in the left atrium results in significantly higher image quality of the pulmonary arteries.*

*• ROI positioning in the left atrium is feasible to perform triple-rule-out CTA.*

## Introduction

In CTA, image quality is strongly influenced by contrast injection timing. As heart rate and cardiac output vary greatly, it is necessary to adapt the time interval between the start of contrast injection and the start of data acquisition in order to achieve optimal contrast filling.

The bolus-tracking technique is widely used for choosing the optimal starting point of data acquisition in CTA. In coronary computed tomography angiography (CCTA), the ROI to monitor contrast inflow is mostly positioned in the ascending aorta (AA), while in triple-rule-out-CTA (TRO-CTA), an examination performed to evaluate the coronary arteries, aorta, and pulmonary arteries, the ROI is usually placed in the left atrium (LA) [1]. The effect of these ROI positions on image quality in CCTA has not been evaluated yet.

The aim of our study was to evaluate the effect of ROI positioning on CCTA image quality, especially with regard to coronary and pulmonary arteries.

## Materials and methods

### Study population and propensity score matching

This study was approved by the local ethics committee, and due to the retrospective evaluation written informed consent was waived. Our radiology information system was searched for patients who underwent CCTA for suspected coronary artery disease between January 2015 and February 2016. Inclusion criteria were the following: patients older than 18 years; intermediate pre-test probability for the presence of coronary artery stenoses as defined by the guideline of the European Society of Cardiology [2]; CCTA performed using the sequential prospective ECG-gated step-and-shoot acquisition technique on a dual-source CT scanner. Exclusion criteria were as follows: CCTA acquisition techniques other than step-and-shoot and incomplete documentation of image data, electrocardiogram, or clinical data. In the selected time period, 352 patients fulfilled the inclusion criteria, including 96 patients with ROI positioning for bolus tracking in the LA and 256 patients in the AA.

Propensity score matching [3] was used in order to minimize the influence of covariates when comparing patients with bolus-tracking ROI in the AA and the LA. This statistical matching technique allowed for the selection of pairs with similar characteristics. It was conducted using R [4] and the “Matching” package for R [5]. Matching criteria were sex, height, body weight, and heart rate. The patients’ sex was matched exactly and the other criteria were matched by the nearest neighbor method [6]. These matching criteria were chosen to minimize differences in heart rate and anatomy. Both heart rate and anatomy are important factors for CCTA image quality due to their potential effects on image blurring and noise.

**Fig. 1:**
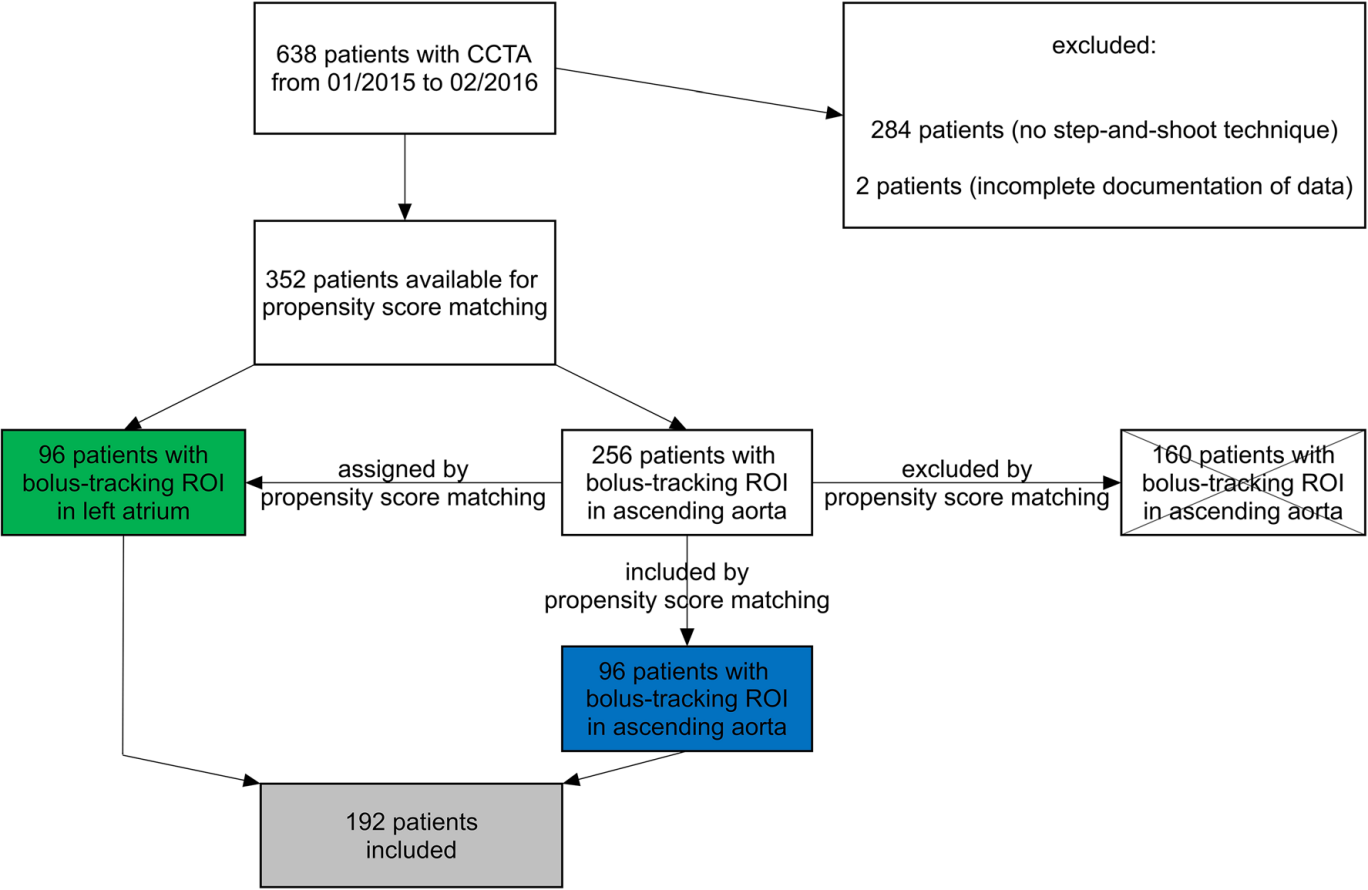
Study design

Based on propensity score matching results, 96 pairs of patients were selected and included in this study (122 males, 70 females, mean age 61 years; 96 patients with bolus-tracking ROI in the LA and 96 patients with bolus-tracking ROI in the AA) (Fig. 1).

In total, 16.1% (31/192) of patients were diagnosed with significant coronary stenosis using CCTA. This included 14.6% (14/96) of patients with bolus tracking in the AA and 17.7% (17/96) of patients with bolus tracking in the LA. A total of 18.2% (35/192) of patients had at least one unevaluable coronary artery segment due to artifacts, including blooming artifacts caused by calcified plaques and motion artifacts. There was one case of pulmonary embolism in our patient cohort.

### Technique

All examinations were performed on a third-generation dual-source CT system (Somatom Force, Siemens Healthineers). Patients were examined in the supine position. An 18-G peripheral venous catheter placed in the right cubital vein was used for i.v. premedication and contrast injection.

In patients with a heart rate higher than 70/min and no contraindications for beta blockers, metoprolol (Beloc®, Recordati Pharma) was applied intravenously to achieve heart rate reduction [7]. Depending on initial heart rate and the patients’ reaction to the initial metoprolol injection, the total dose of metoprolol varied between 2.5 and 15 mg. Two minutes before the start of contrast injection, one push of nitroglycerine pump spray (Nitrolingual®, G Pohl Boskamp) was applied sublingually [8]. Then, 50 ml iopromide (Ultravist 370, Bayer HealthCare) was applied intravenously at a flow rate of 5 ml/s followed by 50 ml saline at the same flow rate using a dual-head injector.

The bolus-tracking technique was used to trigger the start of image acquisition, with ROI placement either in the LA or in the AA. The ROI threshold was 120 HU. The delay time between reaching the threshold and the start of the CCTA acquisition was 5 s. CCTA was performed in inspiration.

In craniocaudal direction, CCTA extended from the tracheal carina to just below the diaphragm. The so-called step-and-shoot technique was used, a prospective ECG-gated, sequential CT technique [9].

Detector collimation was 2 × 192 × 0.6 mm and rotation time was 0.25 s. The tube voltage was adapted by the scanner software CareDose4D (Siemens Healthineers) depending on patient physique. CCTA images were reconstructed with a slice thickness of 0.6 mm using a medium sharp kernel (Bv40) and Advanced Modeled Iterative Reconstruction (ADMIRE [10]) level 3 (out of five possible ADMIRE levels provided by the CT scanner).

### Evaluation of image quality

To evaluate the image quality of coronary and pulmonary arteries, both quantitative measurements (signal-to-noise ratio (SNR)) and qualitative scores were used.

**Fig. 2:**
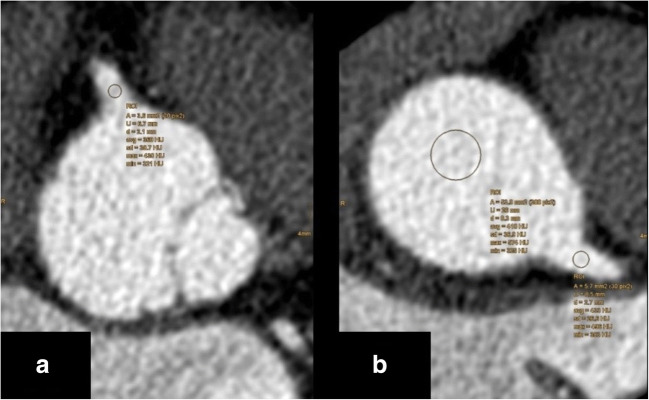
Positions of ROIs for quantitative analysis of coronary arteries (after completion of computed tomography angiography scan). **a** ROI in proximal right coronary artery; **b** ROIs in ascending aorta and left main coronary artery

**Fig. 3:**
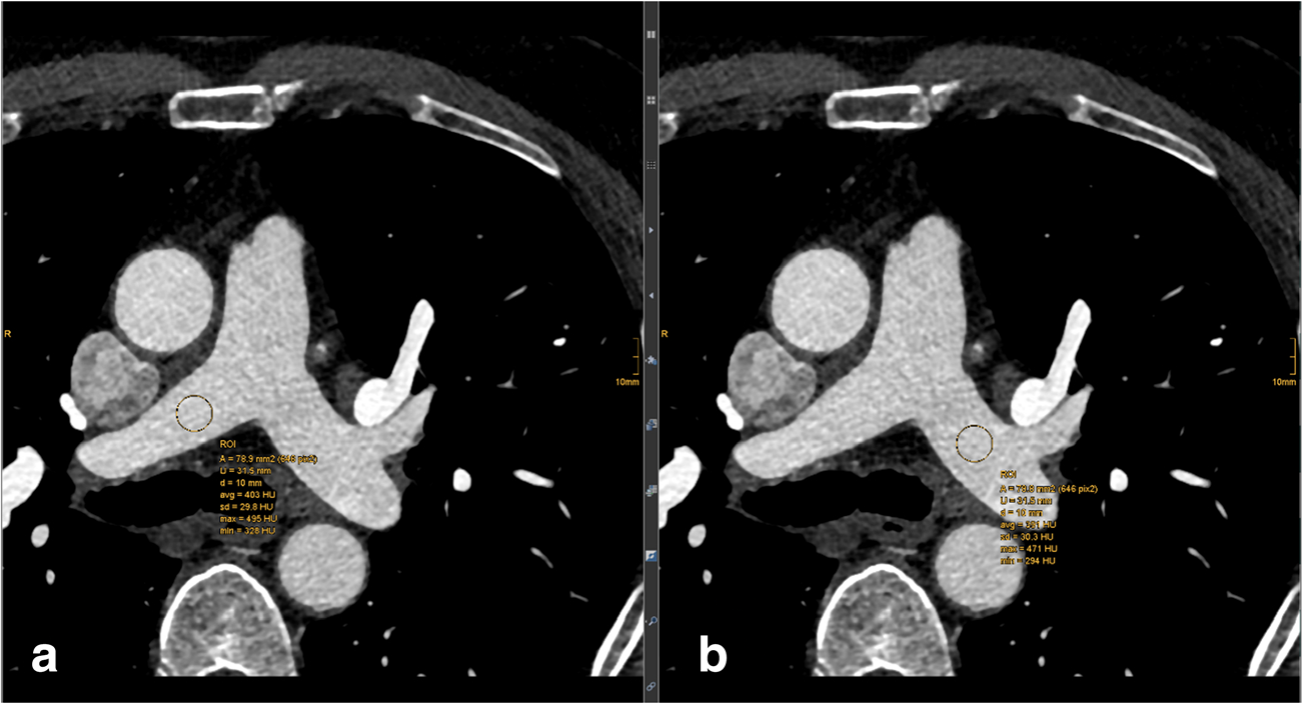
Positions of ROIs for quantitative analysis of pulmonary arteries (after completion of computed tomography angiography scan). **a** ROI in the right pulmonary artery; **b** ROI in the left pulmonary artery

SNR is defined as the quotient of the mean signal intensity and the standard deviation of signal intensity. It was determined in circular ROIs in the ascending aorta, left main coronary artery (LM), proximal right coronary artery (RCA), right pulmonary artery (RPA), and left pulmonary artery (LPA). In the ascending aorta and the pulmonary arteries, the diameter of the ROI was 10 mm and in the coronary arteries it was as large as possible with the exclusion of vessel walls and plaques (Figs. 2, 3).

**Fig. 4:**
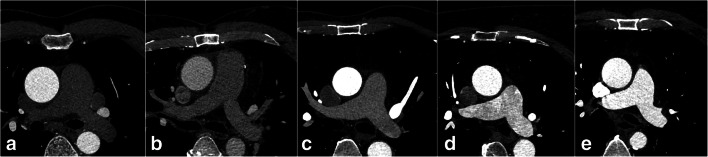
Examples of image quality scores: **a** insufficient (1); **b** poor (2); **c** good (3); **d** very good (4); **e** perfect (5)

For generating the qualitative scores, overall image quality for coronary and pulmonary arteries was assessed independently by two radiologists with 10 and 6 years of experience in CCTA. They were blinded to the position of bolus-tracking ROI. A 5-point Likert scale with the following scores was used: 5 = perfect; 4 = very good; 3 = good; 2 = poor; 1 = insufficient. Vessel wall definition and image noise were considered in the qualitative evaluation of image quality (Fig. 4). In case of disagreement, the total score was decided in consensus.

### Statistics

At first, all variables to be compared were examined for normal distribution by the Shapiro-Wilk test [11]. Since, in most cases, there was no normal distribution, non-parametric tests were used for all comparisons [12].

To prove the success of propensity score matching, the standardized differences in case of height, body weight, and heart rate pre- and post-matching were compared. Based on the recommendation of Austin et al [13], we can assume that there is no significant difference concerning a variable when the standardized difference is lower than 0.1.

As recommended in various articles by Austin [14], Ho et al [15], and Rubin [16], we used tests for paired samples to compare the matched groups of patients.

Thus, signal intensity and SNR were compared using the Wilcoxon test. Because of multiple comparisons, the Bonferroni correction [17] was used to set the significance level. In case of the coronary arteries there are three comparisons; therefore, the significance level was set at *α* = (0.05/3) = 0.0167. In case of the pulmonary arteries, it was set accordingly at *α* = (0.05/2) = 0.025.

Qualitative scores were compared using the Wilcoxon test, too. The significance level was set at *α* = 0.05.

Regarding the qualitative analysis, interrater reliability was evaluated using weighted Cohen’s kappa (*κ*) [18, 19], which was interpreted according to the recommendations of Landis and Koch [20] (*κ* ≤ 0 poor; 0.01–0.20 slight, 0.21–0.40 fair, 0.41–0.60 moderate, 0.61–0.80 substantial, and 0.81–1.00 almost perfect agreement).

Radiation exposure was compared by the Wilcoxon test. Effective doses (in mSv) were calculated by multiplying the dose-length products provided by the scanner with a conversion factor of 18 μSv/mGycm as recommended by Huda et al [21].

The Shapiro-Wilk test, Wilcoxon test, and the calculation of weighted Cohen’s kappa were performed using MedCalc 18.10.02 (MedCalc Software bvba). Diagrams and tables were created using Microsoft Office Excel 2016 (Microsoft Corporation).

## Results

### Propensity score matching

The standardized differences for the comparison of both groups of patients pre-matching were higher than 0.1 in case of height, body weight, and heart rate as well. Post-matching, they were lower than 0.1 in all cases, so we can assume that there was no significant difference concerning these variables in the matched samples [13] (Table 1).

**Table 1:** Standardized differences for the comparison of both groups of patients (bolus-tracking ROI in the left atrium or ascending aorta) pre-/post-matching regarding height, body weight, and heart rate

	Standardized difference	
	Pre-matching	Post-matching
Height	*d* = 0.138	*d* = 0.023
Body weight	*d* = 0.103	*d* = 0.020
Heart rate	*d* = 0.120	*d* = 0.047

### Coronary arteries

#### Quantitative analysis

In the AA, the mean value of signal intensity ± standard deviation (SD) for group A (bolus-tracking ROI in LA) was 531.64 (± 159.18) HU and for group B (bolus-tracking ROI in AA) 512.74 (± 158.27) HU. In the LM, the mean value of signal intensity for group A was 505.08 (± 155.03) HU and for group B 481.31 (± 155.83) HU. In the proximal RCA, the mean value of signal intensity for group A was 516.88 (± 164.81) HU and for group B 455.51 (± 169.88) HU.

**Fig. 5:**
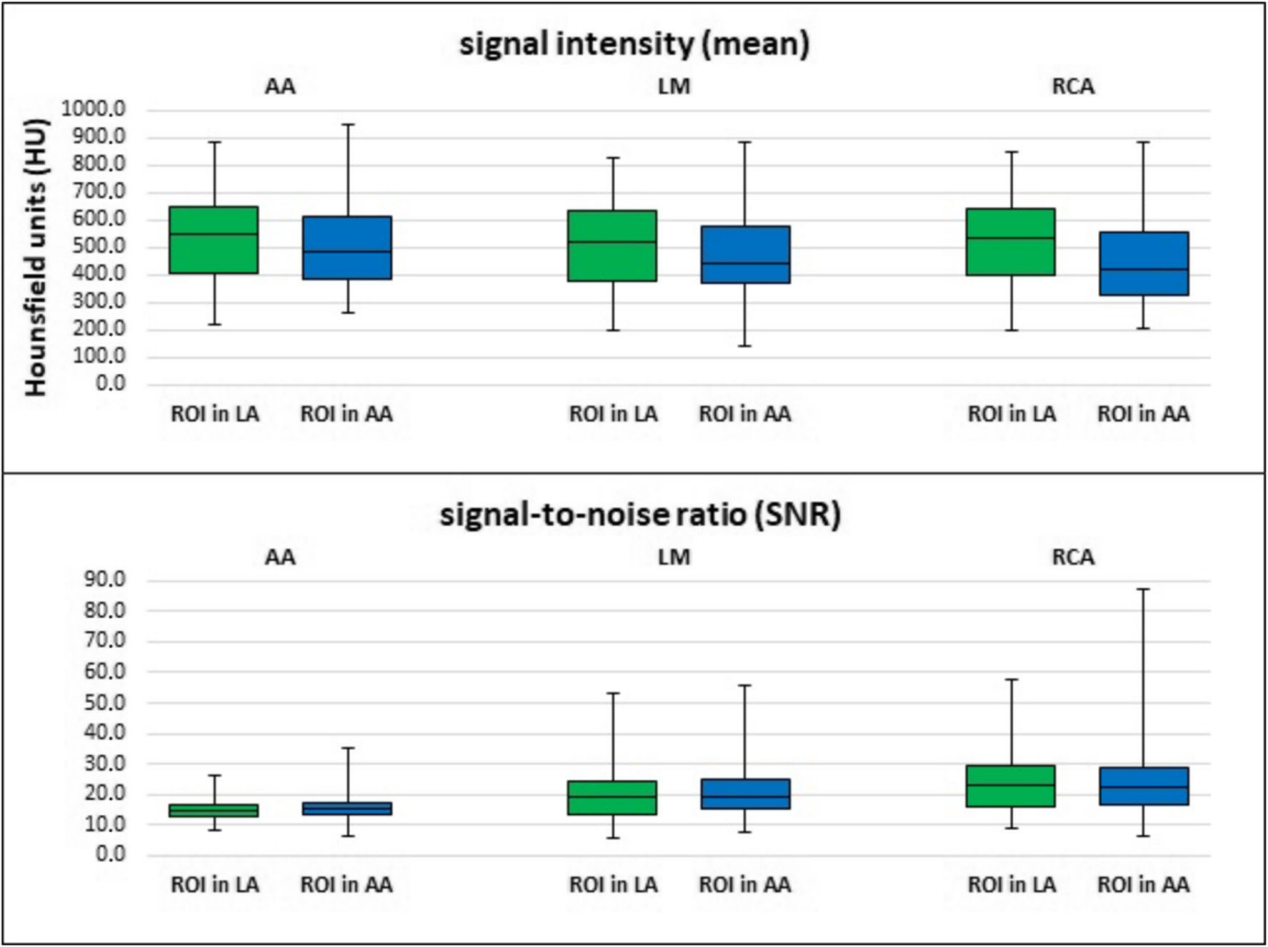
Signal intensities and signal-to-noise ratios in the ascending aorta (AA), left main coronary artery (LM), and proximal right coronary artery (RCA) depending on position of bolus-tracking ROI (left atrium (LA) or ascending aorta (AA)). There was higher signal intensity in the AA (*p* = 0.374), LM (*p* = 0.308), and proximal RCA (*p* = 0.007) for bolus-tracking ROI in the left atrium. There was no significant difference between both groups regarding SNR in the AA (*p* = 0.619), LM (*p* = 0.661), and proximal RCA (*p* = 0.767)

Signal intensity at all three measuring points was higher in group A than in group B. In the RCA, the difference was statistically significant (*p* = 0.007); in the AA (*p* = 0.374) and LM (*p* = 0.308), it was not (Fig. 5).

In the AA, the mean value of SNR ± SD for group A was 14.92 (± 3.75) and for group B 15.46 (± 3.85). In the LM, the mean value of SNR for group A was 19.80 (± 8.36) and for group B 20.30 (± 8.34). In the proximal RCA, the mean value of SNR for group A was 24.34 (± 11.04) and for group B 24.30 (± 12.73).

In summary, there was no significant difference between both patient groups regarding SNR in AA (*p* = 0.619), LM (*p* = 0.661), and proximal RCA as well (*p* = 0.767) (Fig. 5).

#### Qualitative analysis

Out of 96 patients of group A, the image quality was rated 5 in 41 cases (42.7%), 4 in 39 cases (40.6%), 3 in 15 cases (15.6%), and 2 in one case (1.1%); mean qualitative score was 4.25 (± 0.75). Out of 96 patients of group B, the image quality was rated 5 in 44 cases (45.8%), 4 in 36 cases (37.5%), and 3 in 16 cases (16.7%); mean qualitative score was 4.29 (± 0.74).

In summary, image quality scores for coronary arteries did not differ significantly between patients with bolus-tracking ROI in LA and those with bolus-tracking ROI in AA (*p* = 0.672).

### Pulmonary arteries

#### Quantitative analysis

In the RPA, the mean value of signal intensity ± SD for group A (bolus-tracking ROI in LA) was 311.47 (± 159.69) HU and for group B (bolus-tracking ROI in AA) 183.19 (± 114.14) HU. In the LPA, the mean value of signal intensity for group A was 310.98 (± 158.80) HU and for group B 186.32 (± 114.06) HU.

**Fig. 6:**
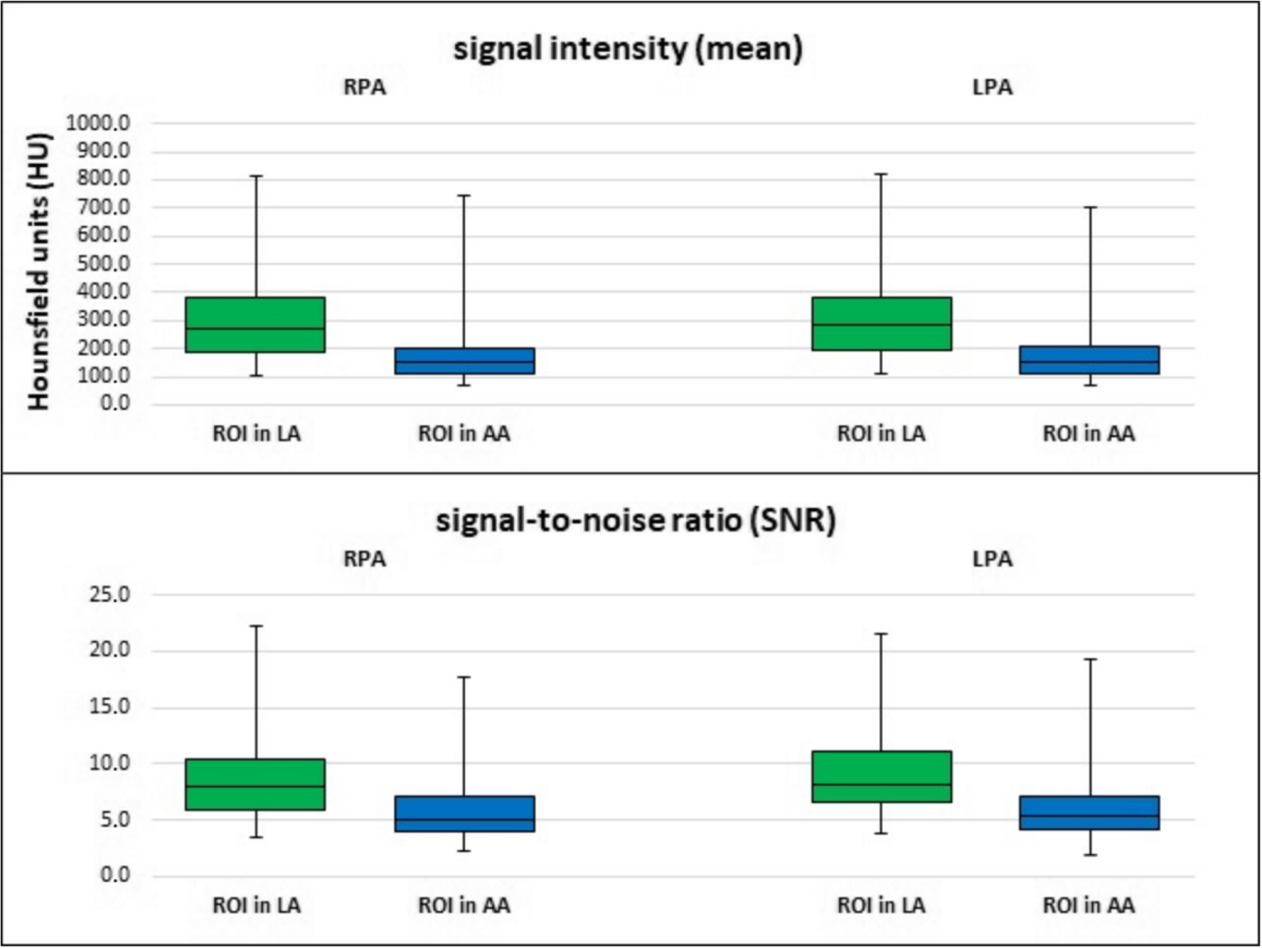
Signal intensities and signal-to-noise ratios in the right pulmonary artery (RPA) and left pulmonary artery (LPA) depending on position of bolus-tracking ROI (left atrium (LA) or ascending aorta (AA)). Signal intensity and SNR in both pulmonary arteries were significantly higher (*p* < 0.001) when bolus tracking in the left atrium was used

Signal intensity in the right (*p* < 0.001) and left (*p* < 0.001) pulmonary artery was significantly higher in group A than in group B (Fig. 6).

In the RPA, the mean value of SNR ± SD for group A was 8.70 (± 3.78) and for group B 5.89 (± 2.98). In the LPA, the mean value of SNR for group A was 9.06 (± 4.03) and for group B 6.25 (± 3.25).

In summary, we found significantly higher SNRs in RPA (*p* < 0.001) and LPA (*p* < 0.001) for bolus-tracking ROI positioning in LA than for bolus-tracking ROI positioning in AA (Fig. 6).

#### Qualitative analysis

Out of 96 patients of group A, the image quality was rated 5 in 47 cases (48.9%), 4 in 18 cases (18.8%), 3 in 19 cases (19.8%), 2 in five cases (5.2%), and 1 in seven cases (7.3%); mean qualitative score was 3.97 (± 1.25). Out of 96 patients of group B, the image quality was rated 5 in eleven cases (11.5%), 4 in four cases (4.2%), 3 in 19 cases (19.8%), 2 in 25 cases (26.0%), and 1 in 37 cases (38.5%); mean qualitative score was 2.24 (± 1.32).

In summary, the image quality scores for bolus-tracking ROI positioning in LA were significantly higher than those for bolus-tracking ROI positioning in AA (*p* < 0.001).

### Interrater reliability

For the qualitative analysis of coronary arteries, both radiologists assigned identical scores in 72.4% (139/192) and different scores in 27.6% (53/192), but they never differed by more than one grade. The resulting weighted kappa was *κ* = 0.654, indicating substantial interrater agreement [20].

For the qualitative analysis of pulmonary arteries, both radiologists assigned identical scores in 74.0% (42/192) and different scores in 26.0% (50/192) and they differed only in one case by more than one grade. The resulting weighted kappa was *κ* = 0.846, indicating almost perfect interrater agreement [20].

### Radiation exposure

The mean dose-length product was 229.23 (± 177.24) mGycm in patients with bolus-tracking ROI in LA and 229.31 (± 141.63) mGycm in patients with bolus-tracking ROI in AA. The corresponding mean effective doses were 4.13 (± 3.19) mSv and 4.13 (± 2.55) mSv [21]. Thus, radiation exposure was almost identical in both groups of patients (*p* = 0.501).

## Discussion

Our study shows that positioning of bolus-tracking ROI in either the LA or the AA does not have a significant impact on coronary artery depiction in CCTA. This result indicates that positioning of the ROI in the AA, which is currently preferred by most radiologists when performing CCTA, is feasible, if coronary artery disease is the main indication for CT.

Our study shows that positioning of the ROI in the LA leads to significantly better image quality within pulmonary arteries and does not adversely affect depiction of the coronary arteries, when compared with conventional positioning in the AA. This implies that as well as achieving superior contrast within the pulmonary arteries, coronary arteries can be reviewed at the same time without loss of image quality by using this ROI position, so it is a valid option when performing a TRO examination. Our results are also in keeping with a study by Ayaram et al [22], which demonstrated that TRO-CTA has similar sensitivity and specificity for coronary stenoses compared with CCTA. This potential gain in relevant diagnostic information comes at the cost of additional radiation exposure due to greater scan length. For example, in a large multicenter, multi-vendor study performed by Burris et al, the median effective radiation dose for TRO-CTA was 9.1 mSv, compared with CCTA at 6.2 mSv [23].

The mean signal intensities in the coronary arteries ranged from 455.51 to 531.64 HU, depending on measuring point and position of bolus-tracking ROI. A study by Becker et al [24] showed an increase in false negative diagnoses of coronary artery stenoses due to overlooked atherosclerotic plaques when signal intensity is higher than 350 HU. Therefore, further reduction of contrast amount or concentration for depiction of coronary arteries seems feasible.

Considering the aforementioned study [24], mean signal intensity in the pulmonary arteries was optimal (311.47/310.98 HU) for group A (bolus-tracking ROI in LA). For group B (bolus-tracking ROI in AA), mean signal intensity was noticeably lower (183.19/186.32 HU), confirming that this ROI position should not be used for TRO-CTA. However, in 31 out of 96 patients in group A (32.3%), qualitative score was 3 or lower. One possible cause is the use of breath-hold in end-inspiration. A recent study has shown that pulmonary CTA should be performed in the resting expiratory position [25]. The image quality of pulmonary artery depiction in CTA can be further improved by using a weight-adapted contrast bolus [26]. Another possible solution would be to increase the injection duration, however at the cost of more contrast agent.

In this study, we used adaptive tube voltage, leading to automatic dose adjustment depending on patients’ physique. Mean radiation exposure was 4.12 mSv. In a study looking at obese patients in CCTA, also using the step-and-shoot technique, mean radiation exposure was 12.34 mSv in the patient group with BMI values comparable with that of our patient group. A possible reason could be the use of constant tube voltage of 120 kV in that study [27]. In contrast, for the also frequently used high-pitch protocol, some studies determined values of around 0.5 mSv [28, 29]. A significant disadvantage of this technique is the increased image noise in patients with higher BMI, so that this protocol is not adequate for patients with coronary heart disease in many cases [30].

In this study, 50 ml of contrast medium was applied for each examination instead of 80 to 100 ml, which was used for examinations with older device generations [31]. Mangold et al examined the influence of BMI on image quality, using the same CT scanner as in our study, but also with the application of higher contrast doses (74.8 ± 14.0 ml) [27]. Nevertheless, higher SNRs were achieved in our study when comparing patient groups within the same BMI range (25–29.9 kg/m^2^). Our results therefore show that 50 ml of contrast medium leads to good coronary image quality and further reduction of contrast volume or concentration might be feasible.

The evaluation of interrater reliability shows substantial agreement of both radiologists regarding the analysis of coronary arteries, which is in keeping with the findings of several other studies [32, 33]. For the analysis of pulmonary arteries, the interrater reliability shows almost perfect agreement. Possible reasons are larger vessel diameters, less pulsation artifacts, and absence of calcifications. Intraobserver reliability was not evaluated, which is a limitation of this study.

Another significant limitation of this study is the lack of correlation with digital subtraction angiography (DSA), which still represents the gold standard for the diagnosis of coronary heart disease. Our study evaluates image quality, but not accuracy of detecting coronary stenoses on CT. Further studies are necessary to compare DSA and CCTA with regard to this. The use of axial slices to measure SNR might also be a limitation. In small vessels like the coronary arteries, the use of orthogonal images could potentially reduce partial volume effects.

Further limitations are the retrospective study design and the use of propensity score matching. Only known influencing factors can be eliminated using this method. And even the considered factors can never agree completely in both patient groups, since the study population is limited.

In conclusion, ROI positioning in the LA leads to good image quality with regard to coronary and pulmonary artery depiction. Therefore, our protocol is feasible to perform TRO-CTA in patients with chest pain.

## Abbreviations and acronyms

AA: Ascending aorta
BMI: Body mass index
CCTA: Coronary computed tomography angiography
LA: Left atrium
LM: Left main coronary artery
LPA: Left pulmonary artery
RCA: Right coronary artery
RPA: Right pulmonary artery
SD: Standard deviation
SNR: Signal-to-noise ratio
TRO-CTA: Triple-rule-out-CTA
